# Prediction-driven active assistance control for lower-limb exoskeletons using multisource information fusion and short-term gait prediction

**DOI:** 10.3389/fbioe.2026.1927528

**Published:** 2026-09-03

**Authors:** Yuanxiang Guo, Yuhui Yang, Wenyu Zhao, Zhijue Huang, Xueshan Gao, Xin Han, Junlin Deng, Peng Zhao

**Affiliations:** 1 Beibu Gulf University, Qinzhou, Guangxi Zhuang, China; 2 Guangxi University of Science and Technology, Liuzhou, Guangxi Zhuang, China; 3 Beijing Institute of Technology, Beijing, China

**Keywords:** human–exoskeleton interaction, lower-limb exoskeleton, multisource information fusion, position-reference control, short-term gait prediction

## Abstract

**Introduction:**

For lower-limb exoskeletons to provide continuous position-reference assistance based on wearer state, the state of human–exoskeleton coupling must be sensed and translated into executable motor commands. However, when prediction outputs are directly fed into low-level controllers without further processing, command discontinuities and increased human–exoskeleton interaction torque may occur because of sensor noise or gait-phase transitions. Multisource information fusion and short-horizon gait prediction are therefore regarded as effective means of addressing this problem.

**Methods:**

In this study, a prediction-driven position-reference control method based on multisource information fusion and a CNN-BiLSTM-GTN model is proposed. Surface electromyography (sEMG), inertial measurement unit (IMU) signals, plantar pressure, joint angles and angular velocities, and human–exoskeleton interaction torques are synchronously collected and organized as sliding-window inputs. In the developed CNN-BiLSTM-GTN model, local dynamic feature extraction by convolutional neural networks, temporal dependency modelling by bidirectional long short-term memory networks, and hip–knee synergistic coupling representation by a graph transformation network are integrated to enable accurate short-term prediction of hip and knee joint-angle trajectories. To make the predicted angles executable, a prediction-driven position-reference assistive strategy is designed. The predicted rolling position references are corrected using measured joint states, gait phase, plantar support state, and interaction torque, thereby generating smooth and bounded joint angle commands.

**Results:**

Experimental results show that the proposed model achieved higher prediction accuracy than the evaluated prediction baselines. The closed-loop experiment further showed that the predicted trajectories could be converted into continuous and bounded position commands with stable tracking.

**Discussion:**

During the evaluated trials, the measured interaction torques remained below the predefined controller intervention threshold.

## 1 Introduction

Lower-limb motor impairment is commonly associated with neurological injury and disease, and affected patients often experience reduced muscle strength, restricted joint motion, and abnormal gait. Rehabilitation therefore requires long-term, repetitive, and task-oriented training. Conventional therapist-assisted rehabilitation can be constrained by therapist availability, training intensity, and cost. Lower-limb rehabilitation exoskeletons can provide repeated and quantifiable mechanical assistance, which supports higher training dosage and more consistent movement practice (Cao et al., 2021; Liu et al., 2020; Wang et al., 2023).

Training with lower-limb exoskeletons has gradually moved from fully passive trajectory following toward active participation and human–exoskeleton cooperation. Body-weight-supported treadmill training can reduce lower-limb loading while supporting high-frequency gait practice (Ma et al., 2021). Overground exoskeleton training further expands the possibility of natural walking and multi-joint coordination (Wu et al., 2018). With the development of compliant actuators and wearable sensors, exoskeletons can adjust assistance according to the user state, limit excessive human–exoskeleton interaction torque, and preserve voluntary contribution (Zhan et al., 2025). Multilevel control strategies have also been used to coordinate assistance levels and trajectory tracking across training stages (Zhang et al., 2022). These studies indicate that continuous active assistance requires more than execution of a preset trajectory. The system must also perceive the user state and adapt assistance to motor ability and gait variation.

Estimating the user’s short-term joint-motion tendency is a key step for continuous active assistance. sEMG reflects neuromuscular activation before and during movement and has been used for lower-limb action recognition and joint-motion prediction (Wang et al., 2024). Temporal convolutional networks, long short-term memory networks, and hybrid architectures have been used to predict continuous joint angles from sEMG or inertial signals (Sun et al., 2024; Zeng et al., 2024). Transformer-based models have further explored synchronous multi-joint prediction (Yu et al., 2024). However, a single signal usually describes only one aspect of the user’s movement state. sEMG provides anticipatory information but is sensitive to electrode placement, fatigue, and inter-subject differences. IMU signals describe limb-segment motion robustly but mainly reflect motion that has already occurred. Plantar pressure is more suitable for detecting foot-contact events and support phases. Existing temporal models also make limited explicit use of structural coupling among the hip, knee, and ankle joints. Therefore, stable continuous joint-angle prediction requires fusion of physiological, kinematic, and contact information, as well as joint temporal and topological modeling.

Accurate short-term joint-angle prediction does not mean that the prediction can be sent directly as a motor command. Sensor noise, gait-phase transitions, and rolling updates of the sliding window can produce abrupt predicted-angle changes. If these predictions are input directly to the low-level controller, they may cause discontinuous commands or increased human–exoskeleton interaction torque (Li et al., 2024; Song J. et al., 2023; Zhu et al., 2023). For lower-limb exoskeletons with a position-control interface, active assistance depends on converting future joint-angle predictions into smooth, bounded position references that respect joint range-of-motion constraints. The controller should also correct the reference using gait phase, plantar support state, and human–exoskeleton interaction torque (Chen et al., 2025; Huang et al., 2022; Xu et al., 2025).

This paper proposes a prediction-driven active assistance control method for lower-limb exoskeleton-assisted walking. The method first fuses sEMG, IMU, plantar pressure, joint angle, joint angular velocity, and interaction torque to build a human–exoskeleton coupled-state input. It then uses a CNN-BiLSTM-GTN network to predict short-term hip and knee angle trends, which provide feedforward motion references for active assistance. Finally, future hip and knee predictions are converted into rolling position references. Measured joint state, gait phase, plantar support state, and interaction torque are used to smooth, saturate, and state-correct the joint commands before they are sent to the low-level motor position controller.

In this manuscript, *active assistance* refers specifically to prediction-informed position-reference tracking on the present exoskeleton; it does not denote torque-based assist-as-needed, impedance, or admittance control. *Prediction-driven control* denotes the high-level process in which the model predicts the next 0.1 s of hip and knee joint angles from a 0.5 s multisource history, after which the predicted sequence is smoothed, bounded, and state-corrected before low-level position execution. The terms *short-term gait prediction* and *short-term joint-motion tendency* refer to future joint-angle prediction from the available sensor signals and do not imply direct measurement of neural motor intention. Human–exoskeleton interaction is quantified by the measured hip and knee interaction torques. In the present controller, a predefined interaction-torque intervention threshold of 25 N⋅m was introduced as a supervisory control parameter. When the measured interaction torque approaches this threshold, the controller adopts a more conservative reference correction. The 25 N⋅m value is an empirical controller-design parameter for the present exoskeleton platform and should not be interpreted as a universal biomechanical or clinical boundary between safe and unsafe human–exoskeleton interaction.

The main contributions are as follows.

1. A multisource information-fusion platform is constructed for lower-limb exoskeleton active assistance. It integrates sEMG, IMU, plantar pressure, joint angle, joint angular velocity, and interaction torque into a human–exoskeleton coupled-state input for gait prediction and active control.
2. A CNN-BiLSTM-GTN short-term gait prediction model is proposed. It combines local feature extraction, bidirectional temporal modeling, and hip-knee topological modeling to predict future hip and knee joint angles.
3. A prediction-driven position-reference active assistance controller is developed. It transforms future joint-angle predictions into smooth and bounded position commands and incorporates gait phase, plantar support, and interaction-torque feedback to adapt the reference correction according to the current human–exoskeleton interaction state.

## 2 System and methods

### 2.1 Overall framework

**FIGURE 1:**
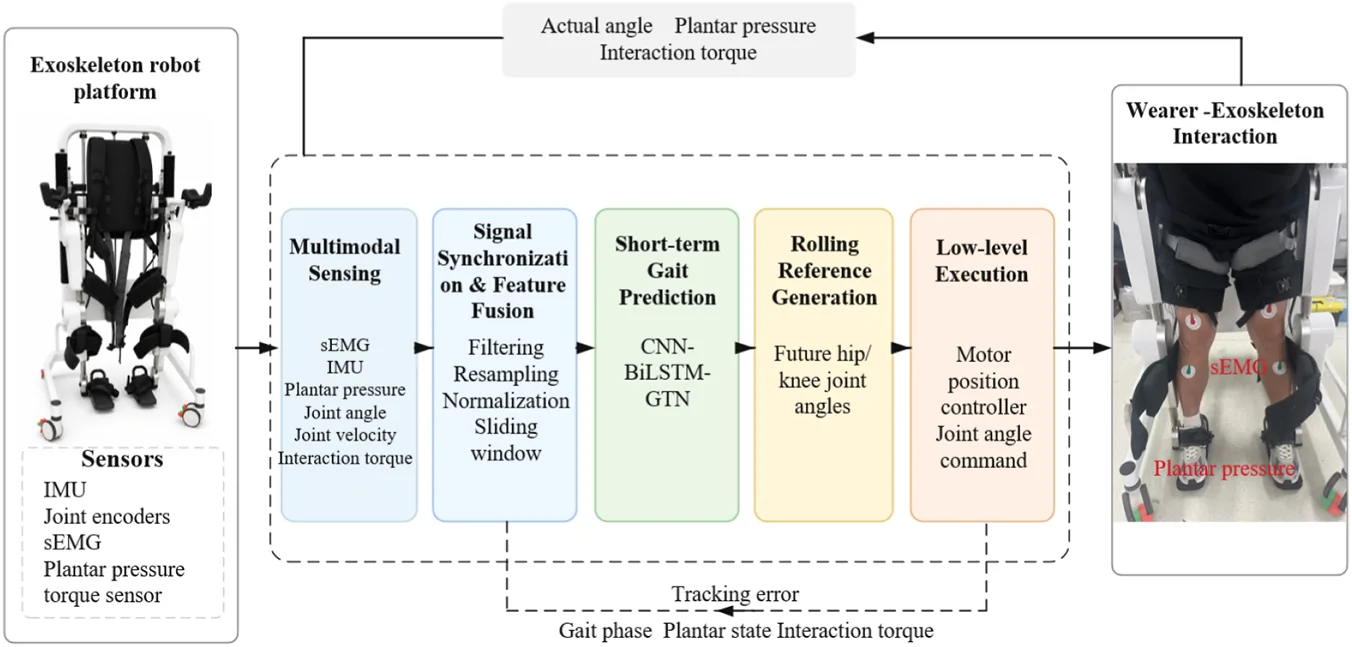
Framework of the lower-limb exoskeleton active assistance control system.

The proposed active assistance control framework is shown in Figure 1. The system contains three modules: multisource information fusion, CNN-BiLSTM-GTN short-term gait prediction, and prediction-driven position-reference control. These modules form a rolling closed loop of state perception, gait prediction, reference generation, position execution, and state feedback. The information-fusion module synchronously acquires and processes sEMG, IMU, plantar pressure, joint state, and interaction torque. The gait-prediction module predicts short-term future hip and knee angles from the historical state window. The active-control module converts the predicted angles into smooth and bounded joint-position commands and sends them to the low-level motor position controller. After exoskeleton actuation, joint state, plantar pressure, and interaction torque are fed back to the system for prediction and control in the next cycle.

### 2.2 Multisource information acquisition

#### 2.2.1 Sensor configuration

**FIGURE 2:**
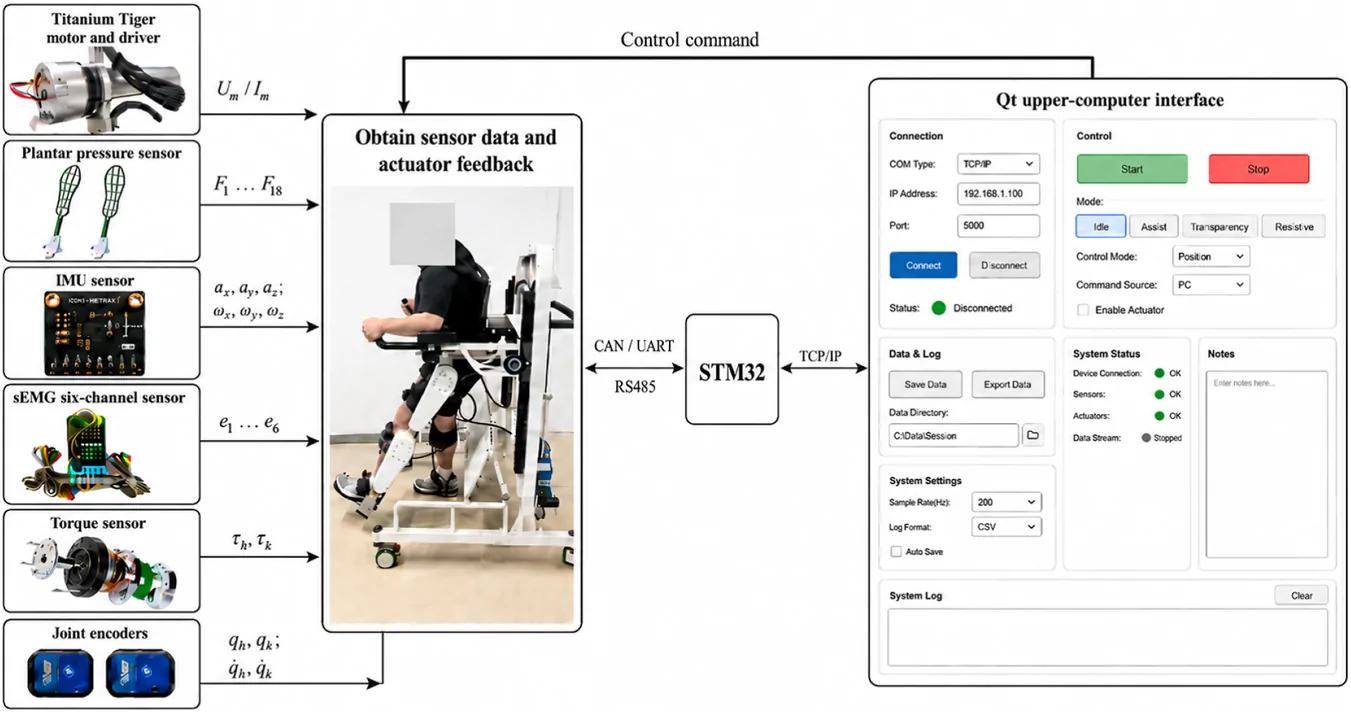
Main components of the multisource information acquisition system.

The multisource acquisition system is shown in Figure 2, and the main hardware components and their specifications are summarized in Table 1. The system consists mainly of joint drive units, joint encoders, torque sensors, plantar-pressure sensors, IMUs, and six-channel sEMG sensors (Jiang et al., 2024). The joint encoders measure hip and knee angles and provide joint angular velocities. The torque sensors record interaction torques between the human and the exoskeleton (Belal et al., 2024). The plantar-pressure sensors identify foot-contact state and gait phase (Song Q. et al., 2023). The IMUs collect lower-limb segment-motion information (Yin et al., 2025). The sEMG sensors acquire muscle-activation signals related to hip and knee flexion and extension (Feng et al., 2025). After synchronization, preprocessing, and sliding-window construction, these signals are used as inputs to the gait-prediction and active-control modules.

**TABLE 1:** Main hardware components and specifications of the multisource sensing and control platform.

Component	Model/Manufacturer	Main specifications
Joint actuator module	JM-TH-CRA-RI, PRO-series harmonic joint module	Integrated frameless torque motor, precision harmonic reducer, servo drive, and 16-bit motor-side absolute encoder; 24–48 V DC; CAN communication
sEMG acquisition module	Six-channel sEMG acquisition module	Six-channel synchronous acquisition; RAW/Envelope modes; configurable sampling rate of 600–10,000 Hz; 5 V DC
IMU	YBX-ICM45686, based on TDK InvenSense ICM-45686	Six-axis MEMS; gyroscope range: ± 15.625 to ± 4000 dps; accelerometer range: ± 2 to ± 32 g; SPI/ ${\text{I}}^{2}$ C; 3.3–5 V
Plantar-pressure sensor	RX-ES42-18	18 independent sensing elements; length: 260 mm; thickness: < 0.3 mm; single-point range: 70 kg; response time: < 20 m; 3.3–5 V
Human–exoskeleton interaction-torque sensor	CHD-NJC	Available measurement range: 5–300 N ⋅ m; safe overload: 120% F.S.; sensitivity: 1.0–2.0 mV/V; zero output: ± 1% F.S.; nonlinearity: ± 0.30% F.S.; hysteresis: ± 0.30% F.S.; repeatability: ± 0.30% F.S.; operating voltage: 5–12 V DC (10 V recommended)

#### 2.2.2 sEMG muscle selection and electrode placement

**FIGURE 3:**
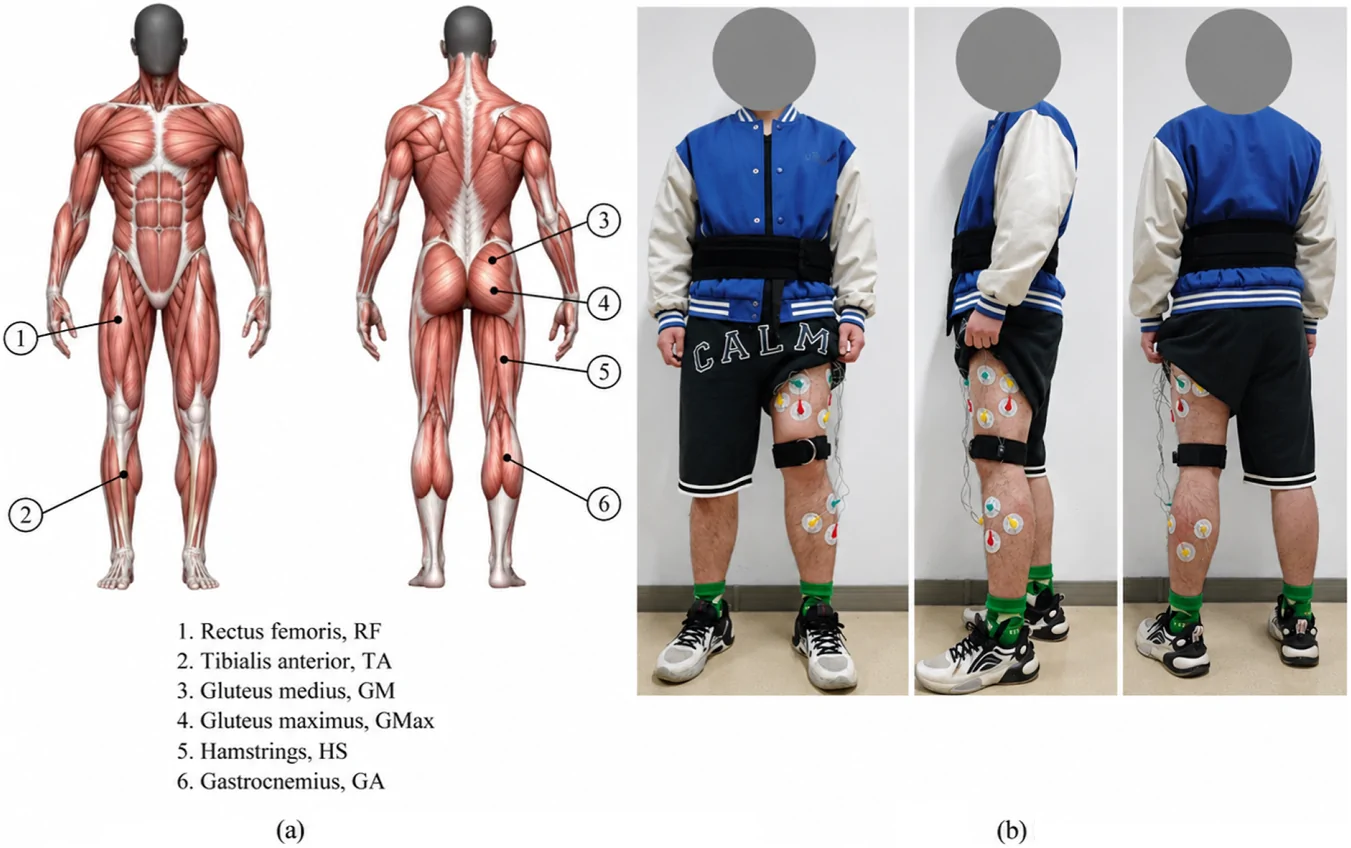
sEMG signal acquisition positions. **(a)** Schematic diagram of sEMG signal acquisition location. **(b)** Experimental diagram of sEMG signal acquisition location.

This study focused on active assistance for sagittal-plane hip and knee flexion and extension. Six lower-limb muscle groups were selected for sEMG acquisition: rectus femoris, tibialis anterior, gluteus medius, gluteus maximus, hamstrings, and gastrocnemius (Lu et al., 2025). The acquisition positions are shown in Figure 3. Before the experiment, the electrode attachment areas were cleaned. Surface electrodes were attached to the muscle belly of each target muscle, and the electrode direction was aligned with the muscle-fiber direction as far as possible. This procedure reduced contact impedance and crosstalk from adjacent muscles (Tu et al., 2024; Yang et al., 2023). The six-channel sEMG signals, together with IMU, plantar-pressure, joint-state, and interaction-torque signals, formed the multisource state input.

### 2.3 Signal synchronization and preprocessing

#### 2.3.1 Signal synchronization

**FIGURE 4:**
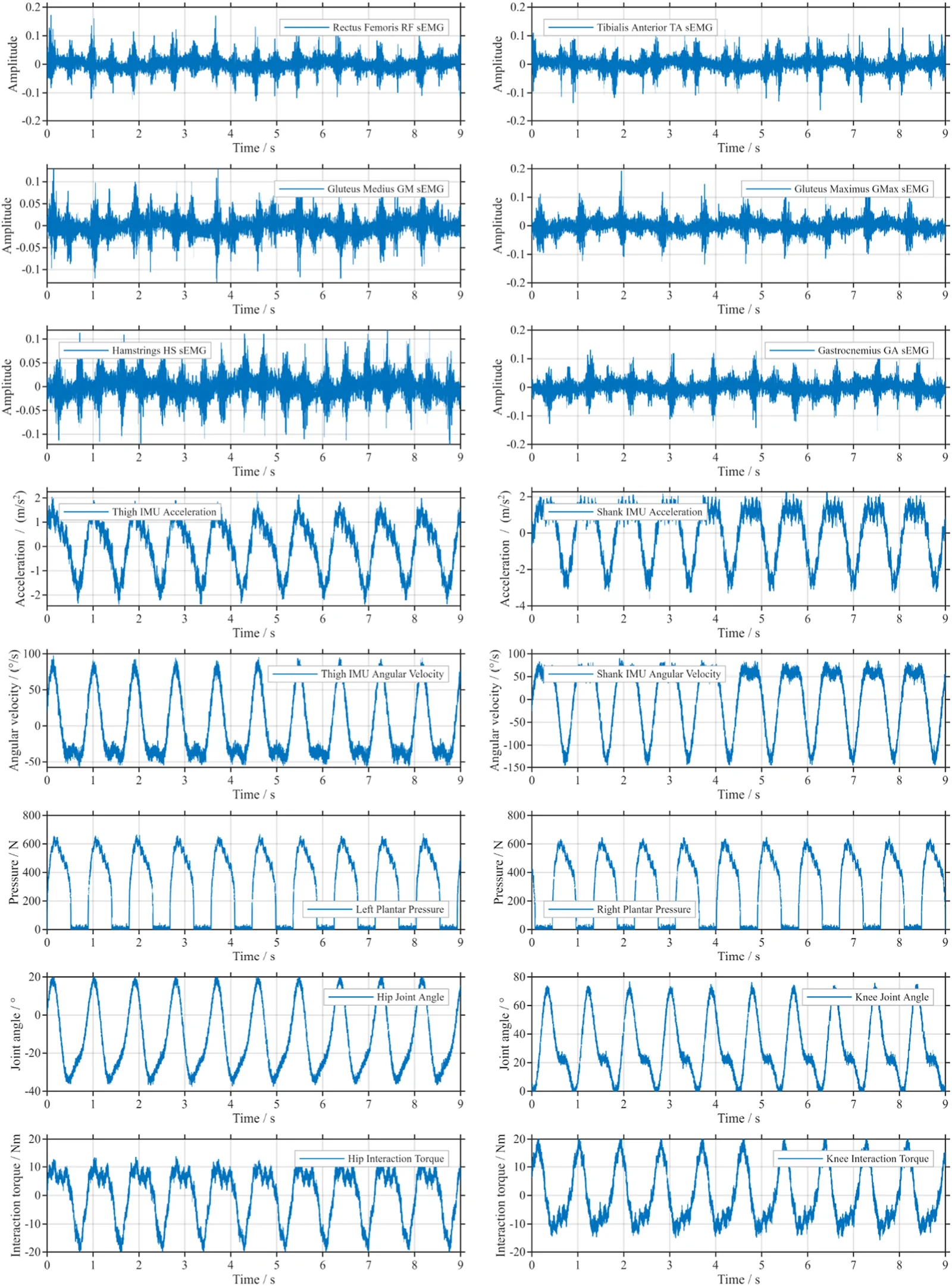
Synchronized raw multisource acquisition data.

Because the sensors had different sampling frequencies and timestamps, all signals were synchronized and resampled before model-input construction. In the experiment, the raw sEMG sampling frequency was 1000 Hz. The IMU, plantar-pressure, joint-angle, and interaction-torque sampling frequencies were 100 Hz. Considering the update cycle of the gait-prediction and active-control modules, all modalities were resampled to 100 Hz, corresponding to a sampling period of 10 m. The multisource state at sampling instant k was represented by Equation 1:

$$s_{k}=\left[x_{k}^{\text{sEMG}},\;x_{k}^{\text{IMU}},\;p_{k},\;\theta_{k},\;{\dot{\theta }}_{k},\;\tau_{k}^{\text{int}}\right],$$ (1)

where $x_{k}^{\text{sEMG}}$ denotes the sEMG signal, $x_{k}^{\text{IMU}}$ denotes the IMU motion information, $p_{k}$ denotes plantar-pressure information, $\theta_{k}$ and ${\dot{\theta }}_{k}$ denote hip and knee joint angles and angular velocities, and $\tau_{k}^{\text{int}}$ denotes interaction-torque information. After synchronization, all modalities were mapped to a unified time axis. The synchronized raw multisource data are shown in Figure 4.

#### 2.3.2 Signal preprocessing

After time synchronization, signals from each modality were filtered, resampled, normalized, and transformed into model features. The specific preprocessing methods are summarized in Table 2.

**TABLE 2:** Preprocessing methods and parameters for different signal modalities.

Signal type	Preprocessing method	Parameters	Output information
sEMG signal	DC-offset removal, band-pass filtering, power-line notch filtering, full-wave rectification, and smoothing	4th-order Butterworth band-pass filter (20–450 Hz); 2nd-order 50-Hz notch filter; full-wave rectification; 50-m moving-average window; resampled from 1000 Hz to 100 Hz	Six-channel EMG envelope signals
IMU signal	Bias correction, low-pass filtering, and resampling	4th-order Butterworth low-pass filter with a 10-Hz cutoff frequency; resampled to 100 Hz	Lower-limb segment acceleration and angular velocity
Plantar-pressure signal	Smoothing, normalization, and threshold detection	5-Point moving-average window (50 m); min–max normalization; normalized contact threshold of 0.10	Left and right plantar support state and gait phase
Joint-angle signal	Low-pass filtering and smoothing	4th-order Butterworth low-pass filter with a 6-Hz cutoff frequency; 5-point moving-average window (50 m)	Hip and knee joint angles
Joint-angular-velocity signal	Angle differentiation followed by smoothing	Central-difference calculation; 5-point moving-average window (50 m)	Hip and knee joint angular velocities
Interaction-torque signal	Low-pass filtering and smoothing	4th-order Butterworth low-pass filter with a 10-Hz cutoff frequency; 5-point moving-average window (50 m)	Hip and knee human–exoskeleton interaction torques

**FIGURE 5:**
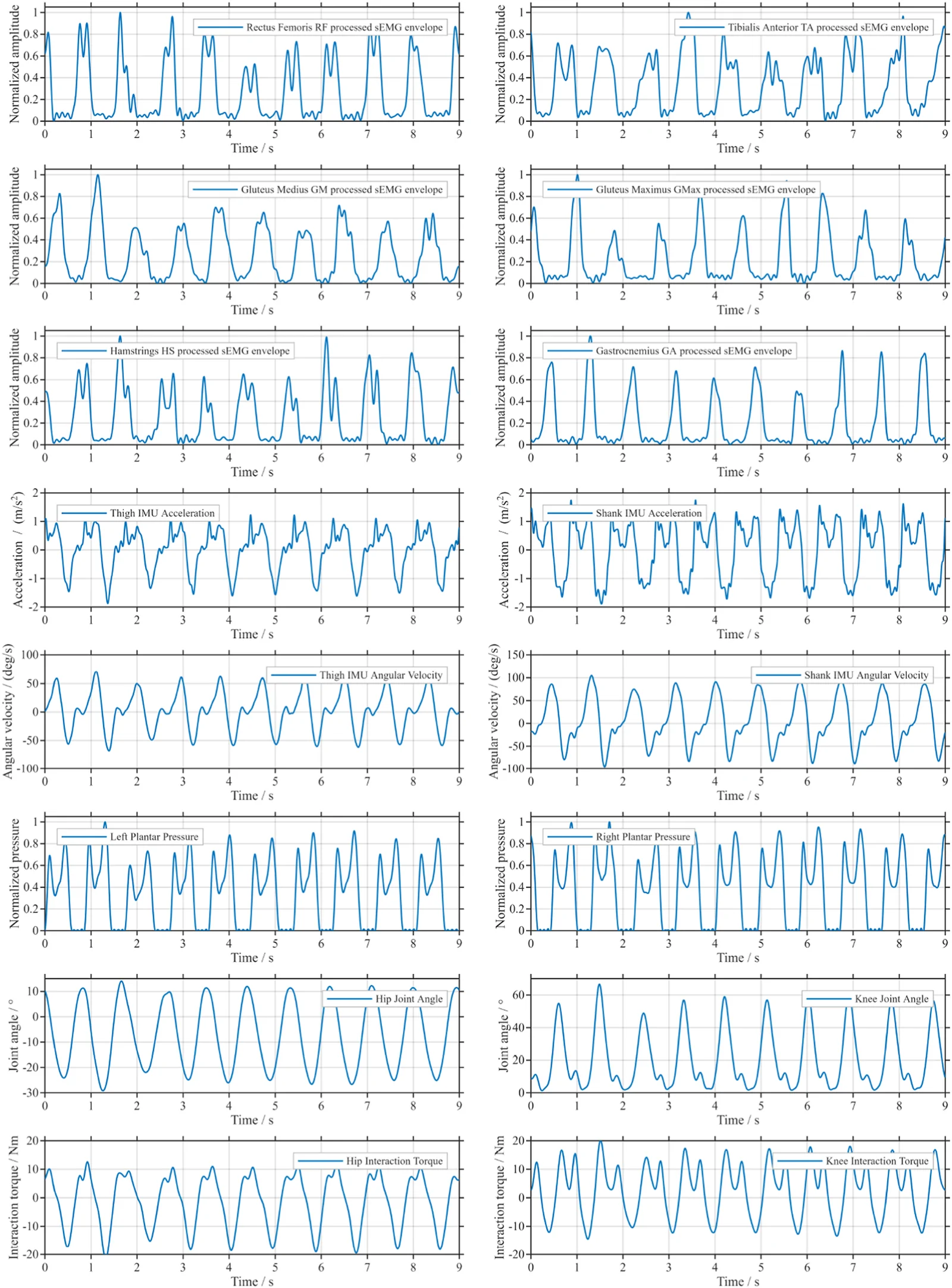
Preprocessed multisource signals.

To eliminate differences in physical dimension and amplitude range among modalities, each synchronized and preprocessed feature was normalized using Equation 2. To prevent information leakage between the training and evaluation subsets, the normalization parameters were calculated exclusively from the training data of each participant. Specifically, $x_{\min}^{\text{train}}$ and $x_{\max}^{\text{train}}$ for each feature were obtained from the corresponding training subset and were then fixed and applied unchanged to the validation and test subsets.

$$\tilde{x}=\frac{x-x_{\min}^{\text{train}}}{x_{\max}^{\text{train}}-x_{\min}^{\text{train}}+\varepsilon },$$ (2)

where x is the raw feature value, $x_{\min}^{\text{train}}$ and $x_{\max}^{\text{train}}$ are the minimum and maximum values calculated exclusively from the training subset, ε is a small constant used to avoid division by zero, and $\tilde{x}$ is the normalized feature. The preprocessed multisource signals are shown in Figure 5. These signals provided a unified time axis and numerical scale for feature extraction and sliding-window input construction.

### 2.4 Feature extraction and multisource input construction

After synchronization, filtering, resampling, and normalization, feature-level fusion was used to construct the input to the CNN-BiLSTM-GTN gait-prediction model. Because different sensors reflect different aspects of the human–exoskeleton state, a single modality cannot fully characterize the state relevant to short-term joint-angle prediction during assisted walking. sEMG, IMU, plantar pressure, joint angle, joint angular velocity, and interaction torque were concatenated at each unified sampling instant to form a multisource fused state vector.

The fused state vector at sampling instant k was defined by Equation 3:

$$z_{k}=\left[e_{k},\;a_{k},\;\omega_{k},\;p_{k},\;\theta_{k},\;{\dot{\theta }}_{k},\;\tau_{k}^{\text{int}}\right],$$ (3)

where $e_{k}$ denotes the six-channel sEMG envelope features, $a_{k}$ and $\omega_{k}$ denote IMU acceleration and angular-velocity features, $p_{k}$ denotes plantar-pressure features, $\theta_{k}$ and ${\dot{\theta }}_{k}$ denote hip and knee angles and angular velocities, and $\tau_{k}^{\text{int}}$ denotes hip and knee interaction torques.

To exploit continuous temporal information in gait signals, the model input was constructed using a sliding window. The previous 0.5 s of multisource fused state was used as the historical input window, and the model predicted hip and knee angle changes over the next 0.1 s. Since all modalities were resampled to 100 Hz, the input-window length was T=50 , and the prediction horizon was H=10 . With sampling instant k as the window endpoint, the multisource input sequence was defined by Equation 4:

$$X_{k}=\left[z_{k-T+1},z_{k-T+2},\ldots ,z_{k}\right]\in R^{T\times D},$$ (4)

where $X_{k}$ is the model-input window, T is the input-window length, and D is the fused feature dimension. The prediction target was the future hip and knee joint-angle sequence over H sampling instants, as shown in Equation 5:

$$Y_{k}=\left[\theta_{k+1},\theta_{k+2},\ldots ,\theta_{k+H}\right],\;\theta_{k+h}={\left[\theta_{k+h}^{\text{hip}},\theta_{k+h}^{\text{knee}}\right]}^{⊤}.$$ (5)

### 2.5 CNN-BiLSTM-GTN short-term gait prediction

**FIGURE 6:**
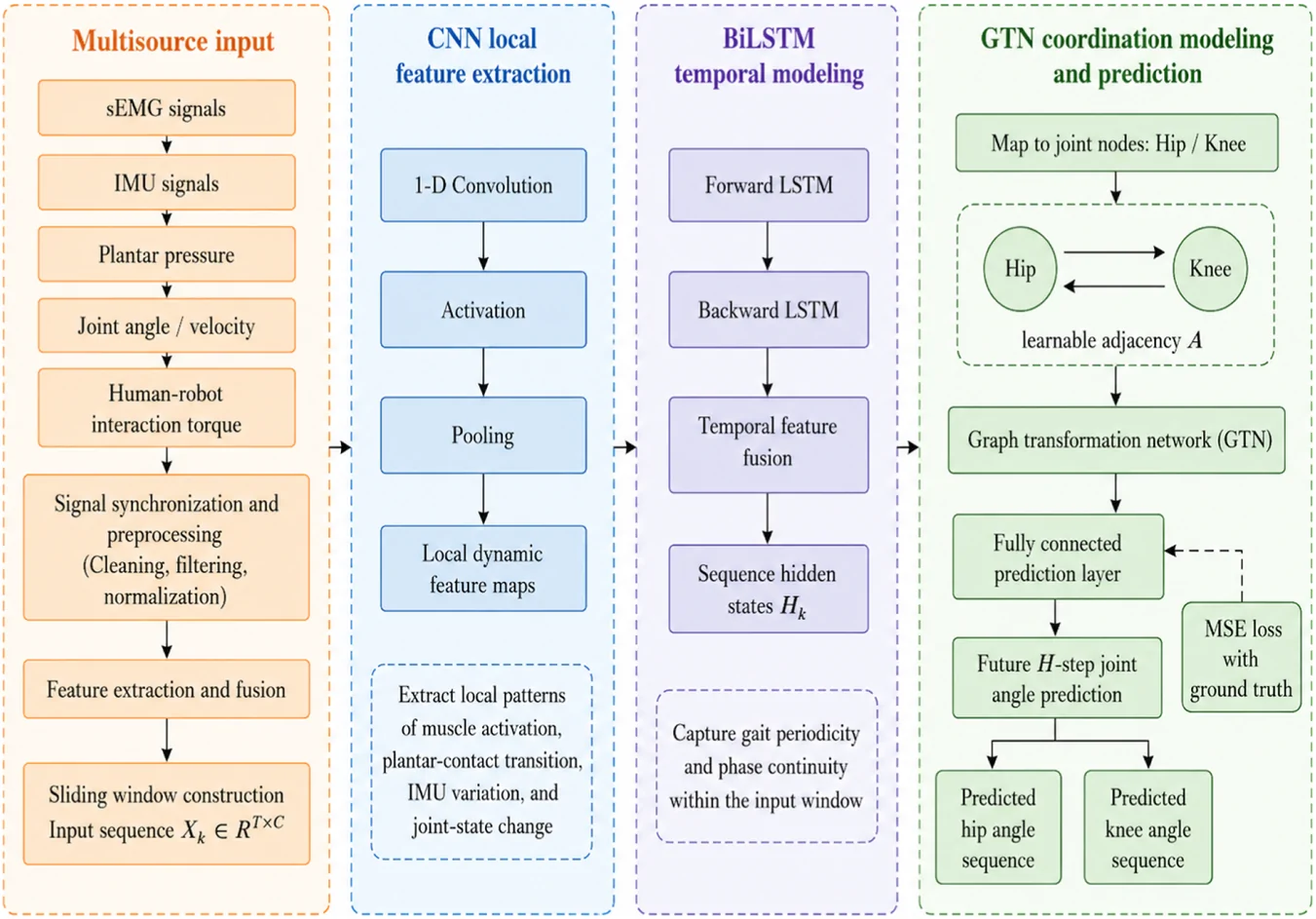
Structure of the CNN-BiLSTM-GTN gait-prediction model.

The CNN-BiLSTM-GTN model used the multisource fused state window as input and output the future short-term hip and knee angles. The model consisted of CNN (Han et al., 2024), BiLSTM (Zhan et al., 2024), and GTN (Yang et al., 2024) components. The CNN extracted local dynamic features, the BiLSTM modeled temporal dependencies, and the GTN represented cooperative coupling between the hip and knee joints (Jin et al., 2025; Zhu et al., 2024). The model structure is shown in Figure 6.

The model included a CNN local feature-extraction module, a BiLSTM temporal-modeling module, a GTN hip-knee cooperative-modeling module, and a fully connected output layer. Its overall mapping was given by Equation 6:

$${\hat{Y}}_{k}=f_{\Theta }\left(X_{k}\right),$$ (6)

where $f_{\Theta }\left(\cdot \right)$ denotes the CNN-BiLSTM-GTN gait-prediction model, Θ denotes trainable parameters, and ${\hat{Y}}_{k}$ denotes the predicted future short-term hip and knee angle sequence.

The model was trained using supervised learning. The mean squared error between predicted and measured joint angles was used as the loss in Equation 7:

$$L=\frac{1}{NHJ}\overset{N}{\underset{n=1}{\sum }}\overset{H}{\underset{h=1}{\sum }}\overset{J}{\underset{j=1}{\sum }}{\left({\hat{\theta }}_{k+h}^{\left(n,j\right)}-\theta_{k+h}^{\left(n,j\right)}\right)}^{2},$$ (7)

where N is the number of training samples, H is the prediction horizon, J=2 is the number of predicted joints, and ${\hat{\theta }}_{k+h}^{\left(n,j\right)}$ and $\theta_{k+h}^{\left(n,j\right)}$ are the predicted and measured angles for sample n , horizon step h , and joint j .

### 2.6 Prediction-driven position-reference active assistance

After future short-term hip and knee angles were obtained, the predicted sequence was used as a rolling position reference. The final joint-angle command was generated by combining the predicted reference with measured joint state, gait phase, plantar support state, and interaction torque.

The use of position-reference control in this study was determined by the low-level control architecture of the experimental exoskeleton, which provides an embedded motor position-control interface. Accordingly, this work focuses on high-level prediction-based reference generation rather than redesigning the actuator-level controller. Torque, impedance, admittance, and assist-as-needed control can regulate physical human–exoskeleton interaction more directly and provide greater intrinsic compliance. In contrast, position control may increase interaction forces when the commanded trajectory deviates from the wearer’s voluntary motion. To mitigate this limitation, the predicted trajectory was not imposed directly as a rigid position command. Instead, the reference was smoothed, bounded, and corrected according to gait phase, plantar-support state, joint angular velocity, and measured interaction torque, together with joint-range and command-rate constraints. These mechanisms improve compatibility with the present position-controlled platform but are not intended to replace intrinsically compliant control strategies.

Suppose the prediction model outputs a future H -step joint-angle sequence at control cycle k . The controller selects a previewed prediction at lead step d as the current prediction reference according to Equation 8:

$$\theta_{k}^{\text{pred}}={\hat{\theta }}_{k+d|k},\;1\leq d\leq H,$$ (8)

where $\theta_{k}^{\text{pred}}$ is the predicted reference angle, d is the lead step, and ${\hat{\theta }}_{k+d|k}$ is the predicted hip and knee angle at future step d generated at cycle k .

Because short-term predictions can be affected by sensor noise, gait-phase transitions, and rolling window updates, first-order smoothing was applied to the prediction reference using Equation 9:

$$\theta_{k}^{\text{ref}}=\alpha \theta_{k-1}^{\text{ref}}+\left(1-\alpha \right)\theta_{k}^{\text{pred}},\;0\leq \alpha <1,$$ (9)

where $\theta_{k}^{\text{ref}}$ is the smoothed rolling reference angle and α is the smoothing coefficient. This process reduces abrupt changes between adjacent prediction windows.

The active-control module then corrected the reference using measured joint angle, joint angular velocity, gait phase, plantar support state, and interaction torque (Zhang et al., 2024). The tracking error was defined by Equation 10:

$$e_{k}^{\theta }=\theta_{k}^{\text{ref}}-\theta_{k},$$ (10)

where $\theta_{k}$ is the measured hip and knee angle. The joint-angle command was generated using Equation 11:

$$\theta_{k}^{\text{cmd}}={sat}_{\left[\theta_{\min},\theta_{\max}\right]}\left(\theta_{k}^{\text{ref}}+K_{p,k}⊙e_{k}^{\theta }-K_{v,k}⊙{\dot{\theta }}_{k}\right),$$ (11)

where $\theta_{k}^{\text{cmd}}$ is the joint-angle command sent to the low-level motor position controller, ${\dot{\theta }}_{k}$ is the measured joint angular velocity, ⊙ denotes elementwise multiplication, and ${sat}_{\left[\theta_{\min},\theta_{\max}\right]}\left(\cdot \right)$ denotes joint-angle saturation. The coefficients $K_{p,k}$ and $K_{v,k}$ are state-dependent position-correction and velocity-suppression gains determined by gait phase, plantar support state, and interaction torque. When plantar support is stable and interaction torque is small, the controller increases the position-correction ratio so that the exoskeleton follows the predicted reference more actively. When the absolute interaction torque increases toward the predefined 25 N⋅m interaction-torque intervention threshold, the controller reduces the position-correction ratio and strengthens velocity suppression so that the predicted position reference is applied more conservatively.

The 25 N⋅m value was empirically selected during controller development as a supervisory intervention parameter for the present exoskeleton platform. It was not quantitatively derived from a statistically characterized baseline interaction-torque distribution, an optimization-based threshold-identification procedure, or a clinically validated biomechanical safety criterion. Its purpose is to define an intervention level at which the controller progressively reduces the position-correction contribution and strengthens velocity suppression when the measured interaction torque increases, rather than continuing to apply the predicted position reference with the same correction intensity. Therefore, the 25 N⋅m value should be interpreted as a platform-specific empirical controller parameter rather than as an optimized or generally applicable interaction-torque threshold.

In this study, the assistance level was not defined as a fixed percentage of body weight or joint torque. Instead, the effective assistance was continuously modulated through the state-dependent gains $K_{p,k}$ and $K_{v,k}$ according to gait phase, plantar-support state, tracking error, joint angular velocity, and interaction torque. The same gain settings and controller constraints were used for all healthy participants, and no normalization based on body mass or maximum voluntary joint torque was applied. Subject-specific adjustment was limited to mechanical fitting and joint alignment.

To ensure command continuity and bounded joint motion (Shushtari et al., 2024), the command was also constrained by joint range of motion and adjacent-cycle rate limits according to Equation 12:

$$\theta_{k}^{\text{cmd}}=clip\left(\theta_{k}^{\text{cmd}},\;\theta_{k-1}^{\text{cmd}}-\Delta \theta_{\max},\;\theta_{k-1}^{\text{cmd}}+\Delta \theta_{\max}\right),$$ (12)

where $\theta_{\min}$ and $\theta_{\max}$ are the lower and upper bounds of allowable hip and knee motion, and $\Delta \theta_{\max}$ is the maximum allowed command change between adjacent control cycles. These constraints reduce abrupt command changes caused by prediction error, sensor noise, or gait-phase transitions.

During closed-loop operation, the active-control module generated joint-angle commands from the current multisource state and prediction result. These commands were sent to the low-level motor position controller. The motor position loop tracked the command and produced hip and knee motion. The executed joint angle, joint angular velocity, plantar pressure, and interaction torque were then fed back to the acquisition module for the next prediction and control cycle.

#### 2.6.1 Initialization and activation of prediction-driven control

At the beginning of each active-control trial, the participant first stood in a neutral posture while the exoskeleton was aligned with the wearer. The prediction-driven controller was not activated immediately. During the initial 0.5-s period, multisource sensor signals were continuously acquired to populate the 50-sample historical input window required by the prediction model. During this initialization period, no prediction-generated gait trajectory was applied.

The initial joint reference and command were initialized using the measured joint configuration, i.e., $\theta_{0}^{\text{ref}}=\theta_{0}^{\text{cmd}}=\theta_{0}$ , to avoid an abrupt mismatch between the wearer and the exoskeleton at controller activation. Once the complete 0.5-s historical window became available, the CNN-BiLSTM-GTN model generated the first 0.1-s-future hip and knee angle sequence. The selected preview point was then introduced into the position-reference controller through the first-order smoothing operation in Equation 9, followed by the state-dependent correction and joint-range saturation in Equation 11 and the adjacent-cycle rate limitation in Equation 12.

Therefore, the first predicted trajectory was not directly imposed on the participant. Prediction-driven control was enabled only after the required historical window had been collected, and the transition from the measured initial joint configuration to the prediction-driven reference was constrained to remain continuous.

## 3 Results

### 3.1 Experimental setup and dataset

Lower-limb exoskeleton-assisted walking experiments were conducted in two separate stages: dataset acquisition for model development and closed-loop active-control validation. Participant information, speed settings, trial numbers, data partitioning, and stage-specific control configurations are listed in Table 3. During the first stage, multisource signals were recorded while the participants performed the prescribed walking tasks with the prediction-driven reference-generation module disabled. Therefore, no CNN-BiLSTM-GTN-generated joint trajectory was applied during the collection of the training, validation, or test data. The measured hip and knee joint angles obtained during these trials were used as the ground-truth prediction targets.

**TABLE 3:** Participant information and experimental protocol.

Item	Description
Number of participants	3 healthy participants
Sex	3 males
Age	24.3±2.5 years
Height	172.6±5.4 cm
Body mass	70.8±6.3 kg
Health status	No lower-limb motor dysfunction
Experimental task	Continuous assisted walking while wearing the lower-limb exoskeleton
Experimental stages	Stage 1: Dataset acquisition for model development; stage 2: Closed-loop active-control validation with the trained model fixed
Speed conditions	Slow, self-selected, and fast gait conditions
Dataset-acquisition trials per participant	5 trials per speed condition, 15 trials in total
Single-trial duration	2 min
Rest interval	1 min between trials
Valid gait cycles	Approximately 900 valid gait cycles
Training-data control configuration	10 Stage-1 trials per participant; prediction-driven reference generation disabled; no CNN-BiLSTM-GTN-generated trajectory applied
Validation-data control configuration	2 Stage-1 trials per participant; prediction-driven reference generation disabled; no CNN-BiLSTM-GTN-generated trajectory applied
Test-data control configuration	3 Stage-1 trials per participant; prediction-driven reference generation disabled; no CNN-BiLSTM-GTN-generated trajectory applied
Training/validation/test ratio	Approximately 66.7%/13.3%/20.0%
Closed-loop active-control data	Collected in stage 2 after deployment of the fixed trained model; used only for control-performance evaluation and not reused for model development or prediction testing
Informed consent	All participants provided written informed consent

After dataset acquisition, the recorded trials of each participant were divided into mutually exclusive training, validation, and test subsets. The CNN-BiLSTM-GTN model was trained and evaluated using these datasets. The trained model was subsequently fixed and deployed in the prediction-driven position-reference controller for the second-stage closed-loop active-control experiments. Data from the closed-loop experiments were used only for control-performance evaluation and were not reused for model training, validation, or prediction testing.

Before prediction-driven control was enabled in each second-stage trial, a 0.5-s initialization period was used to populate the historical input window. Active-control data reported in this section were recorded after completion of this initialization stage.

**FIGURE 7:**
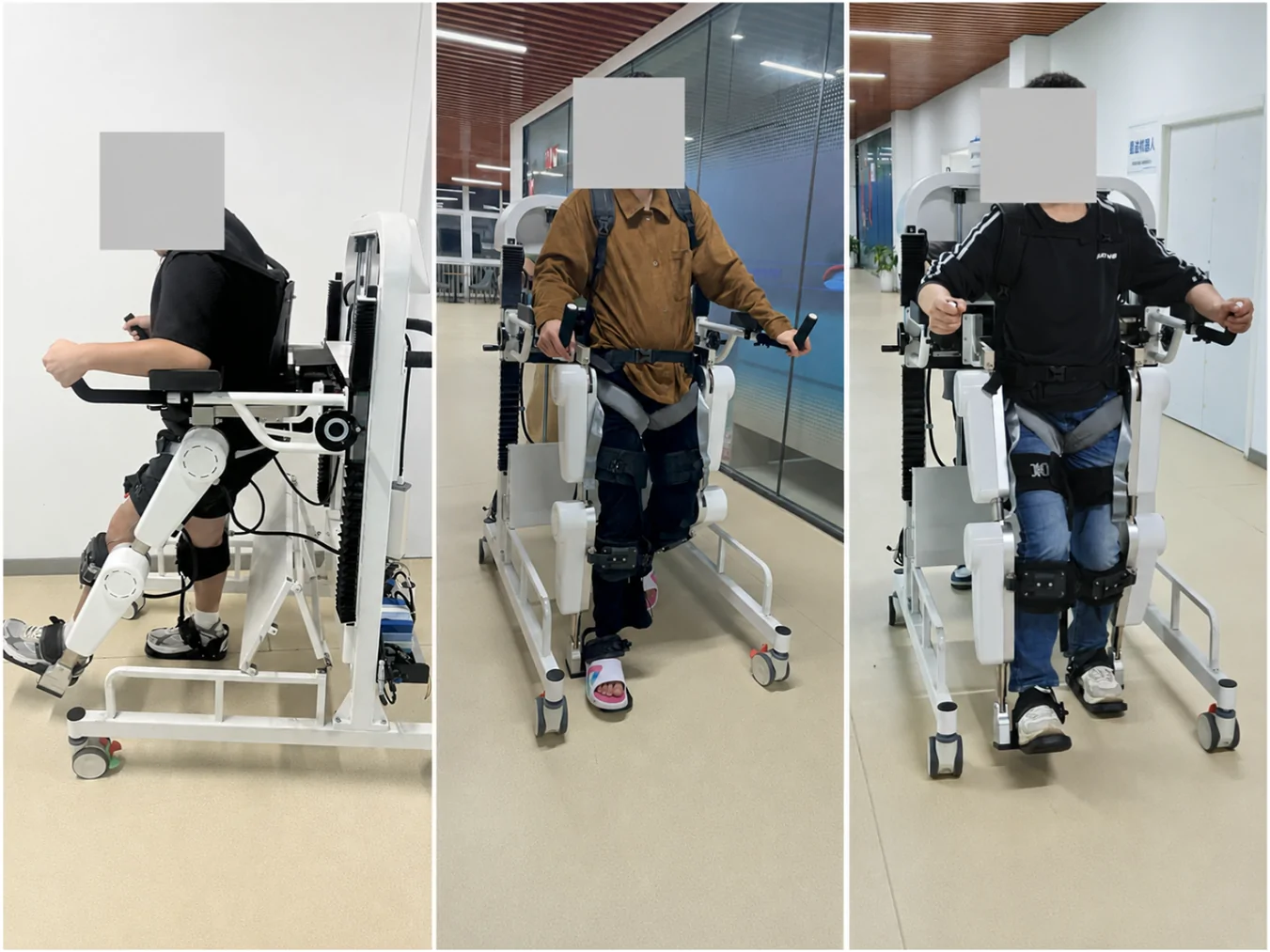
Experimental scene for lower-limb exoskeleton active assisted walking.

During each experiment, raw sEMG was sampled at 1000 Hz, while IMU, plantar-pressure, joint-angle, and interaction-torque signals were sampled at 100 Hz. All modalities were synchronized, resampled to 100 Hz, filtered, normalized, and organized into sliding windows. The experimental scene is shown in Figure 7.

For sample construction, a sliding-window strategy was used to generate model inputs and prediction labels. With a unified sampling frequency of 100 Hz, the input-window length was set to T=50 . Thus, the model used the current and previous 0.5 s of multisource fused state as input. The prediction horizon was set to H=10 , corresponding to the future 0.1 s hip and knee joint angles. The gait-prediction experiment evaluated the short-term joint-angle prediction ability of the CNN-BiLSTM-GTN model. The active-control experiment recorded the predicted reference angle, joint-angle command, measured joint angle, tracking error, and interaction torque to verify the closed-loop feasibility of multisource state perception, short-term gait prediction, and position-reference active control.

### 3.2 Short-term gait-prediction performance

BiLSTM and CNN-BiLSTM without the GTN module were used as comparison models. This comparison evaluated the effects of local feature extraction and hip-knee cooperative modeling on short-term gait-prediction performance. The training parameters are listed in Table 4, and the evaluation metrics were RMSE, MAE, and $R^{2}$ .

**TABLE 4:** Training parameters of the CNN-BiLSTM-GTN model.

Parameter	Value
Optimizer	Adam
Loss function	MSE
Input-window length	50 sampling points
Prediction-window length	10 sampling points
Sampling frequency	100 Hz
Batch size	64
Maximum epochs	100
Initial learning rate	0.001
Gradient threshold	1
Learning-rate drop period	50
Learning-rate drop factor	0.1
Dropout ratio	0.2
Training/validation/test ratio	66.7%/13.3%/20.0%

The model was trained to minimize the error between predicted and measured hip and knee angles. Under the parameter settings in Table 4, the model used 50 consecutive sampling points as the input window and predicted the next 10 sampling points. This setting preserved a short prediction lead while avoiding loss of real-time performance from an excessively long input window.

**FIGURE 8:**
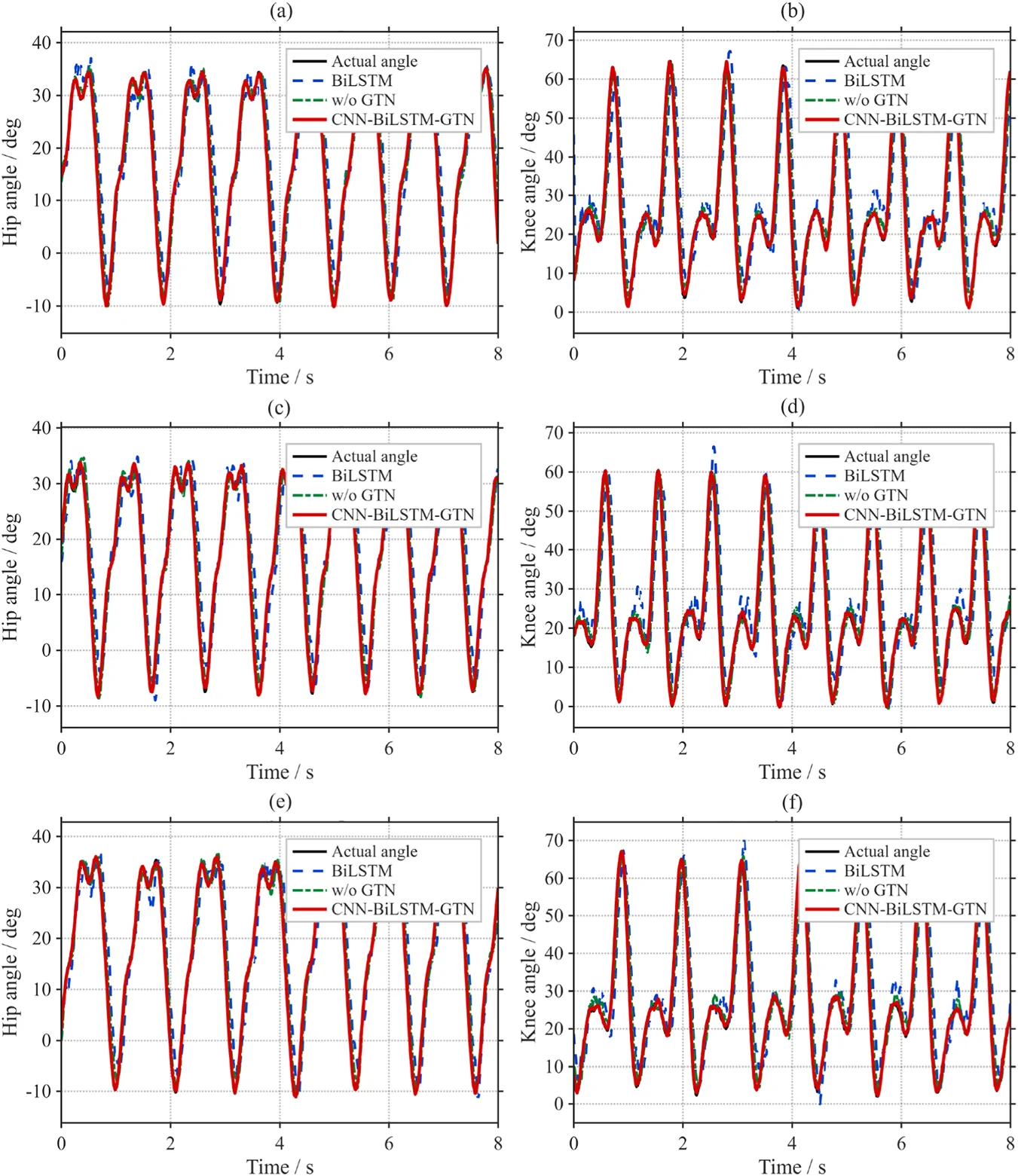
Comparison of short-term hip and knee angle prediction results for different participants. **(a)** Subject 1 hip. **(b)** Subject 1 knee. **(c)** Subject 2 hip. **(d)** Subject 2 knee. **(e)** Subject 3 hip. **(f)** Subject 3 knee.

Figure 8 shows the predicted short-term hip and knee joint angles from the different models. BiLSTM generally followed the periodic joint-angle changes, but clear deviations remained around peaks, valleys, and gait-phase transitions. The CNN-BiLSTM model without GTN improved the predictions, indicating that the CNN-BiLSTM architecture enhanced local dynamic feature extraction and temporal dependency modeling. The proposed CNN-BiLSTM-GTN model produced prediction curves that were closer to the measured curves. It showed smaller deviations at hip flexion-extension peaks, rapid knee flexion, and cycle transitions.

Table 5 shows that the proposed CNN-BiLSTM-GTN model achieved the lowest errors and highest fitting accuracy for both hip and knee prediction. The hip RMSE and MAE were 0.32° and 0.26° , with an $R^{2}$ of 0.9995. The knee RMSE and MAE were 0.45° and 0.36° , with an $R^{2}$ of 0.9993. Compared with BiLSTM, the proposed model reduced hip and knee RMSE by approximately 93.7% and 94.8%, respectively. Compared with CNN-BiLSTM without GTN, it reduced hip and knee RMSE by approximately 86.1% and 89.8%, respectively. These results demonstrate the accuracy and stability of the proposed model for short-term hip and knee joint-motion prediction under the tested walking conditions.

**TABLE 5:** Short-term gait-prediction performance of different models.

Method	Hip RMSE/ °	Hip MAE/ °	Hip $R^{2}$	Knee RMSE/ °	Knee MAE/ °	Knee $R^{2}$
BiLSTM	5.10	3.94	0.870	8.58	6.54	0.739
W/o GTN	2.31	1.82	0.973	4.41	3.32	0.931
Proposed CNN-BiLSTM-GTN	0.32	0.26	0.9995	0.45	0.36	0.9993

### 3.3 Prediction-driven position-reference active control

After validating the short-term gait-prediction model, prediction-driven position-reference active control was tested. The purpose was to evaluate whether predicted angles, after smoothing, saturation, and interaction-torque correction, could be converted into joint-angle commands executable by the low-level position controller. This section focuses on the rolling reference angle, joint-angle command, measured joint angle, tracking error, and interaction torque.

**FIGURE 9:**
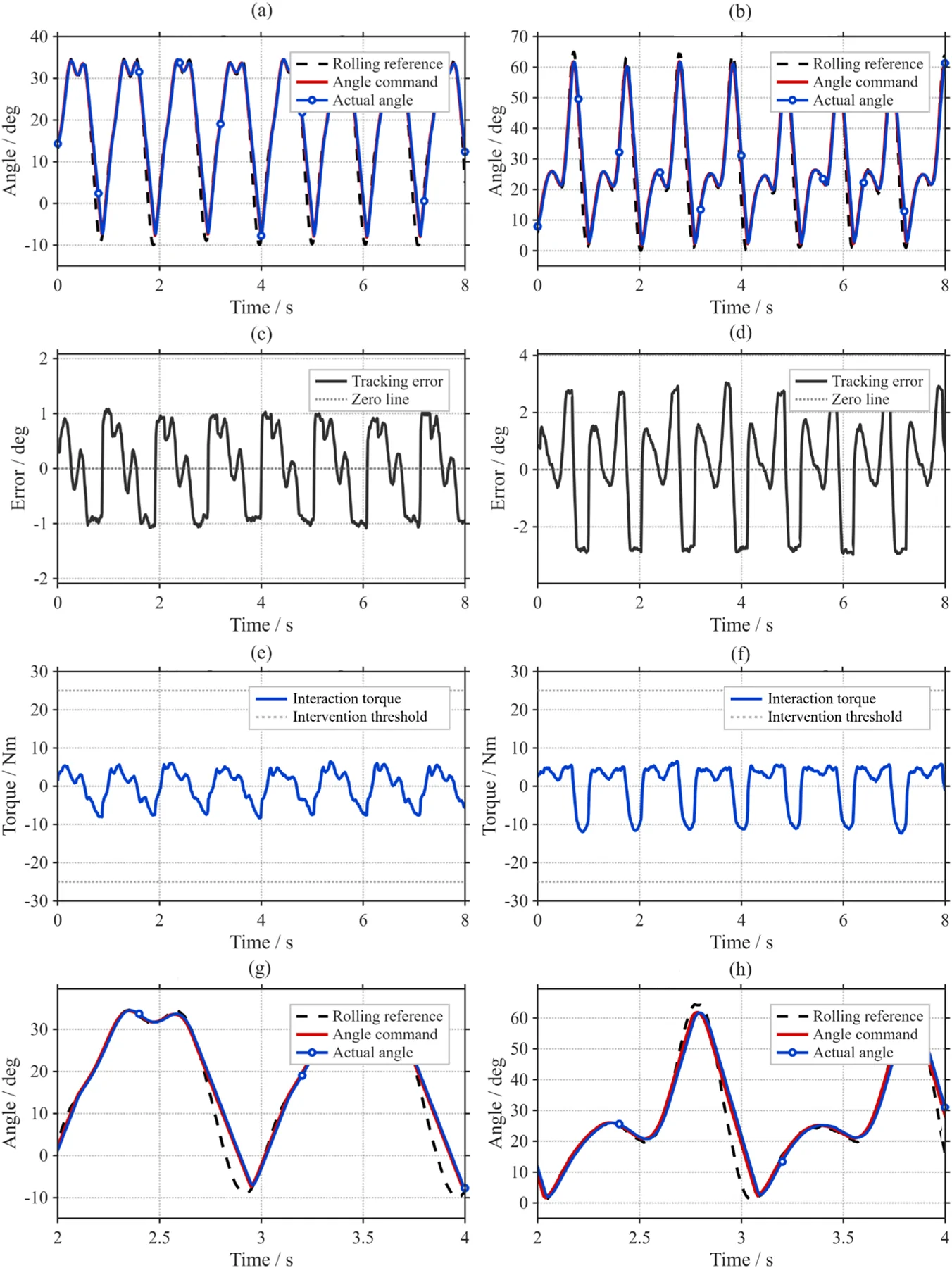
Results of the prediction-driven position-reference active control experiment. **(a)** Hip angle tracking. **(b)** Knee angle tracking. **(c)** Hip tracking error. **(d)** Knee tracking error. **(e)** Hip interaction torque. **(f)** Knee interaction torque. **(g)** Hip zoomed view. **(h)** Knee zoomed view.

Figure 9 shows the prediction-driven active-control results. The smoothed and bounded angle command was more continuous than the rolling prediction reference, indicating that the controller reduced reference jumps caused by adjacent prediction-window updates. The measured joint angles followed the angle commands overall, showing that the corrected predictions could be executed by the low-level position controller. Hip tracking was relatively smooth. The knee showed larger short-term tracking deviations around phase transitions and peaks because of rapid flexion and extension, but the tracking error did not increase continuously.

The active-control performance was quantified using joint tracking error and interaction torque, as shown in Table 6. Both hip and knee joints tracked the prediction-driven angle commands with small errors. The hip tracking RMSE and maximum tracking error were 0.72° and 1.09° , respectively. The knee tracking RMSE and maximum tracking error were 1.80° and 3.05° , respectively. Knee error was higher than hip error, mainly because knee motion has larger angular velocity and stronger nonlinearity during swing-phase flexion and extension. Nevertheless, knee tracking error did not show continuous divergence, indicating that the low-level position controller stably tracked the prediction-driven angle command.

**TABLE 6:** Prediction-driven active assistance control results.

Joint	Tracking RMSE/ °	Maximum tracking error/ °	Peak interaction torque/N ⋅ m	RMS interaction torque/N ⋅ m
Hip	0.72	1.09	8.3	4.1
Knee	1.80	3.05	12.3	6.0

For interaction torque, the peak and RMS values were 8.3 N ⋅ m and 4.1 N ⋅ m at the hip and 12.3 N ⋅ m and 6.0 N ⋅ m at the knee, respectively. During the evaluated prediction-driven closed-loop trials, the measured peak interaction torques remained below the predefined 25 N⋅m controller intervention threshold. These measurements characterize the human–exoskeleton interaction observed during execution of the proposed controller. These observations show that the active-control module generated prediction-driven joint-angle commands while incorporating interaction-torque feedback.

Taken together, Figure 9 and Table 6 demonstrate that the predicted joint trajectories could be converted into continuous and bounded position commands and executed in closed loop on the exoskeleton platform. The observed tracking errors and interaction-torque profiles support the preliminary feasibility and executability of the proposed prediction-to-control chain.

## 4 Discussion

This study proposed an active assistance control method for lower-limb exoskeletons based on multisource information fusion and CNN-BiLSTM-GTN short-term gait prediction. Unlike fixed gait-template following, the method uses sEMG, IMU, plantar pressure, joint angle, joint angular velocity, and interaction torque to construct a human–exoskeleton coupled-state input. It then predicts short-term hip and knee angle trends from the current gait state to provide rolling references for active assistance.

The experimental results showed that the proposed CNN-BiLSTM-GTN model achieved low hip and knee prediction errors. This indicates that multisource fused input can describe gait state more completely than a single sensing modality. sEMG provides feedforward information on muscle activation. IMU and joint-state signals reflect limb-segment motion and measured joint change. Plantar pressure provides gait-phase information. Interaction torque reflects the coupled state between the human and the exoskeleton. Fusing these modalities on a unified time axis helps the model learn relationships among muscle activation, limb motion, foot contact, and joint coordination.

The prediction advantage of CNN-BiLSTM-GTN mainly came from joint modeling of local dynamic features, temporal dependencies, and hip-knee cooperation. The CNN module extracted local variation features from multisource gait signals. The BiLSTM module described temporal evolution within the continuous gait window. The GTN module further introduced structural relationships between the hip and knee joints. Compared with BiLSTM alone or CNN-BiLSTM without GTN, the complete model produced lower hip and knee prediction errors. This result suggests that temporal modeling alone is insufficient to describe multi-joint gait motion, while explicit hip-knee cooperative modeling improves prediction stability.

It should be emphasized that prediction accuracy and assistance effectiveness represent different levels of evaluation. Accurate short-term joint prediction provides a necessary input for prediction-driven reference generation, but it does not by itself demonstrate reduced muscular effort, improved comfort, enhanced gait function, or rehabilitation benefit.

Short-term predicted angles are not suitable as direct motor commands. Predicted values may be affected by sensor noise, gait-phase transitions, and adjacent sliding-window updates. Directly sending them to the low-level position controller could cause abrupt commands or increased human–exoskeleton interaction torque. The proposed controller therefore converted predictions into smooth and bounded rolling position references and applied state-dependent correction using measured joint state, plantar support state, and interaction torque. The active-control experiment showed that the measured hip and knee angles followed the prediction-driven commands, while the measured interaction torques remained below the predefined controller intervention threshold during the evaluated trials. This result supports the preliminary feasibility of the control chain from multisource state perception to short-term gait prediction, position-reference generation, joint tracking, and interaction-torque feedback.

The closed-loop results should be interpreted as a feasibility validation rather than evidence of superiority over alternative control strategies. No direct controller-level comparison with fixed-template trajectory control, torque control, impedance/admittance control, or assist-as-needed control was performed in the present study. Therefore, the reported tracking-error and interaction-torque results demonstrate that the proposed prediction-driven reference-generation strategy can be executed on the current exoskeleton platform, but they do not establish that it provides better assistance quality, compliance, or rehabilitation effectiveness than existing control architectures.

A further consideration concerns the interpretation of the interaction-torque results. The present experimental design did not include a matched controller-level baseline condition specifically intended for quantitative interaction-torque comparison. The present two-stage experimental protocol was designed primarily for prediction-model development followed by closed-loop feasibility validation, rather than as a matched controller-level comparison between baseline operation and prediction-driven control. Therefore, although interaction torque was measured and incorporated into the proposed framework, the current results do not establish whether the proposed strategy reduces or normalizes human–exoskeleton interaction torque relative to baseline operation. The predefined 25 N⋅m intervention threshold should likewise be interpreted as an empirical supervisory controller parameter for the present platform. It was not derived from a statistically characterized baseline torque distribution, an optimization-based threshold-identification procedure, or a clinically validated biomechanical safety criterion. Consequently, the present study does not claim that 25 N⋅m represents an optimal or universally applicable threshold. A dedicated comparative experiment under matched participant, walking-speed, mechanical-configuration, and controller conditions will be required to quantitatively determine whether prediction-driven reference correction reduces or normalizes human–exoskeleton interaction torque. Future evaluation should include peak, RMS, mean-absolute, and high-percentile interaction-torque measures, together with systematic sensitivity analysis of different intervention-threshold settings.

A major limitation of the present study is that all experiments were conducted with healthy participants without lower-limb motor impairment. Therefore, the current results demonstrate only the engineering feasibility of the proposed sensing, prediction, and control pipeline and cannot be generalized directly to clinical rehabilitation populations. The intended future users are individuals who retain partial voluntary lower-limb motor function and measurable voluntary-activation or movement-state signals, such as selected post-stroke patients and individuals with incomplete spinal cord injury. For these users, multisource sensing may help characterize residual short-term joint-motion tendency when any single signal is unreliable, while rolling prediction and interaction-torque feedback could provide state-responsive assistance without relying solely on a fixed gait template. Because neurological impairment may result in altered sEMG patterns, muscle co-contraction, gait asymmetry, restricted joint range of motion, and larger inter-subject variability, prediction models trained on healthy gait data cannot be assumed to maintain the same performance in these populations. Patient-specific calibration and assistance adaptation will therefore be required before clinical application.

Beyond the participant population, this study tested short-term hip and knee angle prediction and position-reference control only during level continuous walking. Prediction stability under different speeds, terrains, long-duration walking, and fatigue states has not been systematically analyzed. Moreover, because the exoskeleton used a low-level position-control interface, the interaction characteristics of the present system were managed mainly through interaction-torque feedback, angle saturation, and command smoothing. Future work should further optimize state-dependent correction, systematically analyze the intervention threshold, and evaluate compliant control alternatives across gait phases.

## 5 Conclusion

This study proposed a prediction-driven active assistance control method for lower-limb exoskeletons based on multisource information fusion and short-term gait prediction. The proposed framework integrates surface electromyography (sEMG), inertial measurement unit (IMU) signals, plantar pressure, joint angles, joint angular velocities, and human–exoskeleton interaction torques to construct a coupled human–exoskeleton state input. On this basis, a CNN-BiLSTM-GTN model was developed to extract local dynamic features, temporal gait dependencies, and hip–knee cooperative relationships, thereby providing short-term joint-angle predictions for active assistance. The predicted angles were further converted into smooth and bounded position references through smoothing, saturation, state-dependent correction, and interaction-torque feedback, allowing the prediction results to be used as executable motor commands rather than direct rigid trajectory inputs.

The proposed architecture establishes an end-to-end control chain from multisource state perception and short-term gait prediction to rolling reference generation, low-level position execution, and interaction-feedback correction. The closed-loop experiments demonstrate that this prediction-to-control chain can be implemented on the physical exoskeleton platform with continuous command generation and bounded tracking errors.

The prediction results verified the effectiveness of the proposed CNN-BiLSTM-GTN model. Compared with the BiLSTM baseline, the proposed model reduced the hip and knee RMSE by approximately 93.7% and 94.8% , respectively. Compared with the CNN-BiLSTM model without the GTN module, the hip and knee RMSE were reduced by approximately 86.1% and 89.8% , respectively. The final prediction errors reached 0.32° for the hip joint and 0.45° for the knee joint, with $R^{2}$ values of 0.9995 and 0.9993, respectively. These results demonstrate that multisource information fusion and hip–knee cooperative modeling significantly improve the accuracy and stability of short-term gait prediction.

The active-control experiment provided preliminary evidence for the executability of the proposed prediction-driven assistance strategy. The hip and knee joints tracked the generated position commands with RMSE values of 0.72° and 1.80° , respectively, and the maximum tracking errors were 1.09° and 3.05° . The observed peak interaction torques were 8.3 N⋅m at the hip and 12.3 N⋅m at the knee during the evaluated prediction-driven closed-loop trials, with both values remaining below the predefined controller intervention threshold. These results demonstrate that the predicted joint trajectories could be converted into continuous and bounded position commands and executed in closed loop on the exoskeleton platform. The observed tracking-error and interaction-torque profiles support the preliminary feasibility and executability of the proposed prediction-to-control chain.

In summary, the proposed method improves short-term gait-prediction accuracy and provides a feasible approach for converting predicted joint motion into continuous and executable position references on the current exoskeleton platform. The present results demonstrate prediction performance and preliminary closed-loop engineering feasibility, but do not establish improved physiological, functional, or clinical assistance outcomes. Further studies incorporating muscular effort, metabolic cost, user comfort, gait-function measures, patient-based evaluation, matched controller-level interaction-torque comparisons, and systematic intervention-threshold analysis are required to assess the actual assistance and rehabilitation effectiveness of the proposed framework.

## Data Availability

The raw data supporting the conclusions of this article will be made available by the authors, without undue reservation.
